# Editorial

**Published:** 1868-01

**Authors:** 


### Editorial.

REPORT ON THE METEOROLOGY, MEDICAL TOPOGRAPHY, AND EPIDEMIC DISEASES OF ILLINOIS.—Dr. R. C. Hamill, of this city, member of the Committee on the above subject for Illinois, made a very interesting report to the American Medical Association at its last Annual Meeting. The report was published in the Transactions of the Association, and a limited number of extra copies furnished to the author in neat pamphlet form. Dr. Hamill is still a member of the Committee of the Association, and is very desirous of obtaining full information concerning the prevalence of all epidemic diseases in this State. Will practitioners throughout the State communicate to him promptly such facts concerning any and all epidemics that come under their observation? If so, they will not only receive due credit therefor, but will very much advance the interests of medical literature and science.

---

Alumni Association of Chicago Medical College.—The first regular Annual Meeting of this Association will be held in the lecture-room of the College in the afternoon of March 3d, 1868. An address will be delivered by the President of the Association, and matters of interest to all will be presented for consideration. In the evening, the members will be cordially entertained at the residence of a member of the College Faculty.

It is earnestly hoped each *alumnus* "will address a letter to the Secretary, on or before the 15th day of February, giving a short history of his professional experience during the preceding year; and stating at length anything of special interest that may have come under his observation."

All *Alumni* of the Institution are earnestly invited to be present to participate in, and contribute to, the interest of the meeting.

S. A. McWILLIAMS, *Sec'y,*
*166 State Street, Chicago.*

---

The Proposed Changes in our Medical College System of Instruction.—We copy the following editorial article from the *Medical Record*, of New York, for Dec. 16th, 1867, for the purpose of showing how the proposed changes are viewed in the best medical periodicals of the East:

### THE PROPOSED CHANGES IN OUR COLLEGE SYSTEM.

A circular has recently been addressed to the various Colleges, asking for a concerted action on their part upon the several prepositions offered by the late Teachers' Convention in Cincinnati. The committee that represents this association, having a more or less permanent character, are perfectly competent to present the matter officially before the different Medical Schools, and in that light this last appeal for reform should undoubtedly be viewed.

They take for their basis of action the resolutions passed not only by the Convention but by the American Medical Association; and as our readers are already familiar with that document in detail, it is unnecessary here to do more than give an outline of the scheme. In brief, then, the changes proposed may be summed up under four heads: First, a positive standard of preliminary education; secondly, a longer time in which to acquire a knowledge of the various branches of Medical Science and Practice; thirdly, a systematic and successive order of studies for the student; and lastly, a certain amount of direct clinical instruction in a public hospital, as part of the senior course.

We have from time to time taken occasion, in our discussion of various matters connected with Medical Education, to urge the adoption of the suggestions therein contained, severally and collectively, and to endorse *in toto* the action of that body. We have not only been of this opinion, but have been ready to go a step or two farther in upholding measures that, under the present system, would seem almost impracticable. But this is by the way.

The objects of the circular which is before us are not only intended to explain away many of the arguments which have seemingly been brought forward by some of the schools against the resolutions referred to, but also to fix a period to the present system of medical instruction. Its first intention has been, to our way of thinking, well carried out, and we have only to hope that its second object will be also attained in an equally satisfactory manner.

In reference to the question of preliminary education we have fully expressed our views, and the plan which we took occasion to suggest was the examination of the students by some competent persons other than the faculty, in order that no plea that any unfairness or partiality could be brought forward. This at the time we believed was practicable, and we are still of the same opinion. There may be some other means adopted to ensure this end, but this matters not, so long as the spirit of the resolution is carried out. The committee have, with some show of policy, merely presented the necessity for the adoption of some feasible means without suggesting anything definite. This is hardly the wisest course that could be pursued, inasmuch as it is advisable to form as a responsible body a distinct platform upon which all points at issue must be directly settled. A failure to distinctly define the manner in which these preliminary examinations should be carried on may still leave the whole question open, a prey to misconceptions and to the temptation of practically ignoring the subject altogether.

On the other proposed changes the committee is more to the point. In regard to the increase in the members of each college faculty good reasons are given. The contemplation is to give four lectures per day to each class throughout the whole college term of six months. We cannot see how this can be objected to by any faculty. The necessary increase of their members will so divide the labor as that each one can perform his duty to himself and to the students. To the supposed decrease in the income of each professor by the adoption of this course, the committee have offered a satisfactory and conclusive answer. The increase in the faculty they assert is amply compensated for pecuniarily by the requirement that each student shall pay full fees for three courses of lectures instead of two. With this the teachers can find no fault, and the student will not be liable to demur, especially when no choice is left him in order that he may be sure of his diploma in the end. The increased expense for the whole term will not be felt any more for the third year than for the two previous ones, while in the end the increased outlay on his part brings him a richer premium in the allowance of extra time to benefit by the instruction which he receives.

The section referring to the branches to be taught during each term was simply for the purpose of naming a definite course to be pursued by each college, so that such a uniformity in the general system might be obtained as would allow students of different classes to transfer themselves to different colleges without occasioning confusion.

In order to obviate embarrassment in making the change from the present system of college instruction to the one proposed, the committee prudently suggest "that all students who have so nearly completed their period of study at the time fixed for making the change that an attendance on an additional course would render them eligible to graduation, should be allowed to complete their course by attending the Senior department under the new arrangement; while all who are in the first half of their period of study should be subject to the new arrangement in full." This proposition is an eminently proper and practical one, and should effectually do away with the principal obstacle to the speedy carrying out of the new plan.

The time set for the inauguration of these changes is during the winter term of 1868-9, and it strikes us that there is ample opportunity offered to all the colleges to discuss the matter by answers to the following questions by the committee:—

1st. Do your faculty, together with the governing authority of your College, approve of the several propositions as a whole?

2d. If you do not approve of the plan of revision as a whole, what changes would you suggest?

3d. If you approve of the plan as a whole, or of all its essential features, will your College be ready to adopt it practically, and issue your Annual Announcement for the Chicago term of 1868-9, in accordance therewith; provided all the principal Medical Colleges in this country (or at least those in the cities of Boston, New York, Philadelphia, Baltimore, Richmond, Charleston, New Orleans, Louisville, Cincinnati, St. Louis, Chicago, Buffalo, and Albany) will agree to do the same at the same time?

These answers are to be addressed to the Chairman, Dr. N. S. Davis, of Chicago. We hope that every respectable Medical College in the land will consider it a bounden duty to give the subjects referred to the fullest consideration, and be prepared when the time comes to agree, if not entirely upon the plan as set forth, at least sufficiently so to insure that uniformity of action which shall crowd out every element but honorable competition and an earnest desire to raise the standard of Medical Education.

There is no reason why a response should not be received from every College by the committee before the first of March next, when opportunities would be given to call another Convention of Medical Teachers, and have a report presented to them in due form.

We hope the last paragraph here quoted, will attract the special attention of the several college faculties. The questions proposed by the committee merit a fair and explicit answer; and it is exceedingly desirable that these answers should be in possession of the committee by the time specified above.

---

Dr. Roberts Bartholow has been appointed Professor of Materia Medica and Therapeutics in the Medical College of Ohio.—*Medical Record.*

A Friend has sent us the following rare specimen of medical opinion and prescription:—

It will do either for a laugh, or for a sample of human ignorance and gullibility.—[Ed.

Sylvester Green Co., Wis.

Dec. 9, 1867.

Mrs Catherine Reutschler your lungs be somewhat Weak they Don't feel Natural, the lungs Are not organicly Diseased your liver very torpid the liver Don't secrete its Portion of bile it throws the bile into the Gall Ducks & that Don't receive it As it should therefore it Passes into the Stomach & blood Affecting your stomach Much At times you have Distress to your Stomach At times that the World Don't know of the Digestive organs Are Weak the food Lies hard in your Stomach At times the Affection of the liver causes Pain between your Soulders At times the Stomach Affects the Nerves the nerves Affect your head At times your Kidneys are Weak the Glands that Run to the Neck of the Bladder Are irrtated this Affects the Water At times the Weakness of the Kidneys Causes weakness in your back & such A Dull Heavy tried feeling in your Hips & Limbs At times

Prescription.

one oz of Colombo Root half oz of Rheubarb half of extract of Dandelion one fourth of A oz of Aloes Put All of these in one quart of Whiskey take one table spoonfull A half hour before breakfast.

half oz of Peruvian bark one fourth of A oz of balm Gilead buds Put these in one Gill of Alcohol let it stand twenty four hours then Add one Pint of Maderia Wine take one table spoonfull A half hour before Dinner & Supper the White of one hens egg one table spoonfull of loaf sugar one table spoonfull of fourth Proof brandy beat All well together & take this quantity in the Middle of the Afternoon take A Pill of Asafoedita the Size of A Common Pea in the Middle of the forenoon Eat Light food Drink Dandelion Coffee with your Meals scorch the Roots & Make it As other

Coffee Mix equal Parts of Mullen leaves & Pulverised Cinnamon bark together & smoke it shortly After each Meal the Mullen leaves that Grow Near the Ground Are good now. for A Linament one oz oil of origenum half of Sweet oil half oz of Spirits hartshorn half oz of Laudanum

Put All of these together Put it on your forehead & temples Morning noon & night & smell of it At the same time

Continue these Medieines Six Weeks then you Are to be be seen Again you will be better if you Do As Directed.

examination & Prescription
by Dr J Dodge.

---

### CORRECTION.

Editor of Examiner.—In the article on "Tetrachloride of Carbon," of last month, there is an error, implying that Dr. Sherman's experience in giving anæsthetics amounts to "some thousands of cases." It should read "above a thousand of cases." E. ANDREWS, M.D.

---

Money Receipts till Dec. 25, 1867.—Drs. O. B. Ormsby, Murphysboro, Ill., $3; G. Wheeler Jones, Danville, Ill., 3; W. R. Atkinson, Mt. Hope, Wis., 3; M. W. Wilcox, Mattoon, Ill., 5; Q. M. Triplett, Little Rock, Ill., 3; J. H. Hollister, Chicago, Ill., 3; L. J. Burrows, Janesville, Wis., 3; W. H. Price, Danby, Ill., 3; S. Wickersham, Chicago, Ill., 3; S. K. Faulkner, Whitesville, Mo., 3; Alonzo Clark, Beloit, Wis., 3; G. D. Maxson, Nile, N.Y., 3; John Lewis, Ogden, Ind., 3; P. J. Morris, Jericho, 3.

---

Death from Chloroform.—A death from chloroform is reported in Chicago. The cause is referred to simple asphyxia from the toxic effects of the article, although portions of cracker, of which the patient had partaken shortly before the administration of the anæsthetic, were found in one of the bronchial tubes. The chloroform was pure, and but an ounce was administered. Every means to restore the patient was resorted to without avail. *Medical Record.*

---

The packing of bottles, filled or empty, is now performed more safely, closely, and rapidly than heretofore, by means of India-rubber rings slipped over them. The rings cost only once, and can remain on the bottle as long as it lasts.—*Med. Gax.*

MORTALITY REPORT FOR THE MONTH OF NOVEMBER:—

The monthly report is as follows:—

CAUSES OF DEATH.

| | | | |
|---|---|---|---|
| Accidents, | 11 | Heart, valvular disease of, | 2 |
| Apoplexy, | 2 | Hemorrhage, umbilical, | 1 |
| Avota, aneurism of, | 1 | Hepatitis, | 1 |
| Augina, pectori, | 1 | Hydrocephalus, | 2 |
| Asphyxia, chloroform, | 1 | Hydrocephalis, acute, | 1 |
| Abscess, psoas, | 1 | Intemperance, | 1 |
| Birth, premature | 13 | Intemperance and Morphine, | 1 |
| " still | 27 | Insanity, | 1 |
| Brain Congestion of, | 5 | Inanition, | 6 |
| " Inflammation of, | 6 | Jaundice, | 2 |
| Bowels, inflammation of | 1 | Kidneys, disease of, | 1 |
| Bowels, obstruction of | 1 | Liver, cancer of, | 1 |
| Bright's Disease, | 1 | Laryngitis, | 3 |
| Bronchitis, | 8 | Lungs, congestion of | 1 |
| Cancer, | 1 | Lungs, hemorrhage of, | 2 |
| Cancer, encephaloid, | 1 | Lumpus, | 1 |
| Cancer, calcum, | 1 | Measles, | 8 |
| Cancer, knee-joint, | 1 | Meningitis, | 3 |
| Catarrhal Inflammation, | 1 | Meningitis, Cerebro-Spinal, | 4 |
| Croup, | 18 | Meningitis, Tubercular | 1 |
| Croup, diphtheric, | 1 | Morbus Coxarius, | 1 |
| Cholera Infantine, | 4 | Old Age, | 5 |
| Cholera-Morbus, | 1 | Peritonitis, | 3 |
| Convulsions, | 27 | Peritonitis, puerperal, | 1 |
| Cyamosis, | 2 | Pericarditis, acute, | 1 |
| Debility, | 7 | Paralysis, | 2 |
| Delirium Tremens, | 2 | Paralysis, agotous, | 1 |
| Diarrhœa, | 3 | Phthisis Pulmonalis, | 34 |
| Diarrhœa, cronic, | 1 | Pneumonia, | 16 |
| Diphtheria, | 9 | Pneumonia, typhoid, | 3 |
| Dropsy, | 4 | Scrofula, | 1 |
| Dysentery, chronic, | 1 | Small-Pox, | 18 |
| Dysentery, | 4 | Suicide, | 2 |
| Enterites, | 1 | Stomach, cancer of | 3 |
| Epilepsy, | 3 | Tabes Mesenterica, | 9 |
| Erysipelas, | 2 | Tetanus, | 1 |
| Exhaustion, | 1 | Teething, | 5 |
| Fever, congestive, | 1 | Throat, disease of, | 1 |
| Fever, typhus, | 4 | Trismus, | 1 |
| Fever, typhoid, | 26 | Urennia, | 1 |
| Fever, scarlet, | 3 | Whooping-Cough, | 1 |
| Fever, ship, | 1 | Wound, | 1 |
| Heart Disease, | 4 | | |
| Heart, hypertrophy of, | 1 | Total, | 370 |

NATIVITIES.

| | | | | | |
|---|---|---|---|---|---|
| Chicago, | 195 | England, | 8 | Scotland, | 4 |
| Other parts U. S., | 72 | France, | 3 | Sweden, | 3 |
| Austria, | 1 | Germany, | 42 | Switzerland, | 1 |
| Belgium, | 0 | Holland, | 1 | Wales, | 1 |
| Bohemia, | 2 | Ireland, | 37 | Poland, | 1 |
| Canada, | 2 | Norway, | 11 | | |
| Denmark, | 3 | Prussia, | 10 | Total, | 370 |

Ages of the Deceased.—Under 5 years, 156; over 5 and under 10 years, 17; over 10 and under 20, 11; over 20 and under 30, 40; over 30 and under 40, 43; over 40 and under 50, 20; over 50 and under 60, 14; over 60 and under 70, 12: over 70 and under 80, 9; over 80 and under 90, 6; 90 and under 100, 1; premature births, 13; still born, 28; unknown, 0. Total, 370.

COMPARISON.

| | |
|---|---|
| Deaths in November, 1867, | 370 |
| Deaths in November, 1866, | 382 |
| Decrease, | 12 |
| Deaths in October, 1867, | 428 |
| Decrease in November, | 58 |

SEXES.

Males, 204 | Females, 166 | Total, 370

COLOR.

Colored, 7 | White, 363 | Total, 370

MARRIED AND SINGLE.

Single, 263 | Married, 107 | Total, 370

DEATHS IN EACH WARD.

The following table gives the average of deaths in each ward, on the basis of the population of 1866:

| Ward. | Mortality. | Pop. in 1866. | One death in | Ward. | Mortality. | Pop. in 1866. | One death in |
|---|---|---|---|---|---|---|---|
| 1 | 8 | 9,668 | 1,208 1-2 | 14 | 19 | 12,108 | 636 1-5 |
| 2 | 24 | 12,985 | 541 1-4 | 15 | 30 | 15,766 | 525 1-5 |
| 3 | 20 | 15,738 | 786 18-20 | 16 | 18 | 14,912 | 789 5-9 |
| 4 | 14 | 10,884 | 777 6-14 | County hos., | 14 | | |
| 5 | 18 | 9,610 | 533 16-18 | River, | 1 | | |
| 6 | 23 | 10,580 | 460 | Bridewell, | 2 | | |
| 7 | 39 | 18,755 | 483 1-2 | Home of the | | | |
| 8 | 16 | 10,429 | 651 4-5 | Friendl's, | 0 | | |
| 9 | 30 | 13,940 | 464 | Marine hos., | 3 | | |
| 10 | 9 | 11,416 | 1,266 1-3 | Mercy hos., | 7 | | |
| 11 | 24 | 12,924 | 537 4 5 | Orph. asy., | 2 | | |
| 12 | 31 | 12,695 | 409 1-2 | St. Joseph hos. | 1 | | |
| 13 | 17 | 8,188 | 481 3-5 | Lake Mich., | 0 | | |

Total, 370

---

The Prize Essay on Physical Longevity—by American Popular Life Insurance Company. This company offered some time since a prize of $500 for the best essay upon this subject. Two communications of the number presented were so meritorious, and it was so hard to decide the question of superiority that a prize was given to each. The successful authors were Dr. J. H. Griscom, of New York, and Dr. J. V. C. Smith, of Boston.—*Medical Record.*

---

A Grandmother at Twenty-eight.—An old practitioner who is giving the reminiscences of his medical experience in the *Gazette Medicales de Lyon*, enumerates among them the case of a young girl in his vacinity, who became the mother of a healthy child at the age of fourteen. The child was a girl, and in her turn became a mother at fourteen years of age. The young grandmother, he says, is still hard at work in her village, and is very proud of her title to a uniqne frame as a grandmamma at twenty-eight.—*Medical Record.*

---

Dr. E. S. Connor accepts the Chair of Medical Chemistry in the Medical College of Ohio, vice Dr. Roberts Bartholow, resigned.—*Medical Record.*

---

LITHOTOMY AND LITHOTRITY.—In the *Nashville Journal*, for July, Dr. Paul F. Eve gives a tabular statement of 90 operations for stone performed by him, during a period of twenty-five years. In 78 of these, the bilateral operation was performed, resulting in 70 speedy cures and 8 deaths. Of the latter, Dr. Eve thinks four should not have been operated upon, as they were very unfavorable cases; a fifth died of dysentery when the wound had nearly healed. The ages were: 45 under 10 years; 24 between 10 and 30 years; 3 between 30 and 50 years; 6 over 50 years; and the average less than 16 years. The remaining 12 cases were of a mixed character, viz., lateral and high operations, vaginal section and lithotrity. Three of the number died. Dr. Eve attributes the death in three cases to want of skill, (for during the war, the operation had to be performed without proper instruments,) and adds: "I honestly believe, that of these cases of lithotomy not more than two or three can be put down as fatal cases under ordinary circumstances." In contrast to these results of *lithotomy* are the records of 70 cases of *lithotrity* performed by Mr. Henry Thompson, of London, and published in the *Lancet.* Only four deaths occurred in these cases, and one was from organic disease of the kidney, after complete relief from the calculi and recovery from the effects of the operation. Very few of the patients were under 50 years of age, and the majority were over 60 years. The average age of all the patients was 62 years. Many of them were in circumstances of health which rendered interference very hazardous, and the greatest care and attention were necessary. Cases were not selected. The most striking fact in this contrast is the great difference in the average age, the lithotomy cases averaging less than 16 years and the lithotrity cases about 62 years, and the records of the latter fully justify Mr. Thompson in heading his article, "Proofs that lithotrity is an eminently successful operation."—*Pacific Med. & Surg. Journal.*

A Doctor's Bill in the Reign of William III.—In the diary of Sir Thomas Rokeby, justice in the Court of Common Pleas in the reign of William III., just published, occurs the worthy valetudinarian's doctor's bill for only two months, October and November, 1697:—"Purging pills, 2s.; leeches, 6d.; aperitive ingredients, 1s. 6d.; hystericke water, 2s.; a purging bolus, 1s. 6d.; purging pills, 1s.; Gascan powder, 4s.; vermifuge pills, a box, 3s. 4d.; a purging bolus, 1s. 6d.; purging pills, 1s. cephalick drops, 2s. 6d.; an hysterick julep, 3s. 6d.; hysterick pills, 85, 6s. 8d.; a vomitive potion, 2s. 6d.; a stomattick cordial, 2s.; a cordial potion, 1s. 8d.; vomitive salts, 3 doses 1s 6d.; the hysterick julep, 3s. 6d.; Mithridate, 1s.; the vomitive potion, 2s. 6d.; vomitive salts 1s. 6d.; the hysterick pills, 6s. 8d.; the hysterick julep, 3s. 6d.; sal-armoniac, 6d.—2*l*. 17. 10." Spite of this drenching, to which he had subject himself, he lived to the age of 67.—*British Medical Journal.*

---

Experiments made by Drs. Rigner and Rickards on the effect of alcohol on men and animals, go to show that the temperature of the body falls nearly as fast after the use of alcohol in cases sufficient to produce intoxication, as after death itself. facility with which drunkards freeze to death, is explained by this fact. Dr. Jolly declares, that an increasing tendancy towards mental disease has been generated by the increasing consumption of spirits. Official reports show, that the abuse of alcohol accounts for one-fifth of the insanity in France.—*Med. Gazette.*

---

New Treatment for Tænia.—A novel method of expelling tape-worm is adopted by Dr. Lortet. While recognizing the value of oil of male fern, and other anthelmintics, he is, nevertheless, looking after something better to give in those intractable cases in which the usual remedies may have been vainly used. Dr. Lortet has administered, in a few cases, sulphuric ether, which acts upon the worm as it does upon man, that is, renders it insensible. Shortly after the exhibition of the ether, a mild purgative is given. The plan is to give five drachms of ether at a dose, and to follow it in two hours by an ounce of castor oil. The worm is discharged without causing pain, entire or almost so, and always with the cephalic end intact. Though but few patients have been subjected to this treatment, yet its uniform success, even in two instances where other means had failed, renders it worthy of notice.—*Pacific Med. & Surg. Jour.*

